# NK cell based immunotherapy against oral squamous cell carcinoma

**DOI:** 10.3389/fimmu.2024.1440764

**Published:** 2024-08-13

**Authors:** Ying Zhang, Jianming Xie, Haoran Wu, Jinhui Huang, Danna Zheng, Shaotong Wang, Xueqiang Jia, Zongzhong He, Ying Gong, Linling Ju, Qiurong Sun

**Affiliations:** ^1^ Department of Stomatology, The First Affiliated Hospital of Gannan Medical University, Ganzhou, Jiangxi, China; ^2^ Department of Otolaryngology & Head and Neck Surgery, Anyuan People’s hospital, Ganzhou, China; ^3^ Southern Medical University, Guangzhou, Guangdong, China; ^4^ Department of Transfusion Medicine, General Hospital of Southern Theatre Command, Guangzhou, Guangdong, China; ^5^ Department of Laboratory Medicine, Nanfang Hospital, Southern Medical University, Guangzhou, Guangdong, China; ^6^ Medical School of Nantong University, Nantong Third People’s Hospital, Affiliated Nantong Hospital 3 of Nantong University, Nantong, China

**Keywords:** oral squamous cell carcinoma, immunotherapy, natural killer cells, tumor microenvironment, CAR-NK

## Abstract

Oral squamous cell carcinoma (OSCC), a major subtype of head and neck cancers, presents significant challenges due to its aggressive feature and limited therapeutic efficacy of conventional treatments. In response to these challenges, Natural Killer (NK) cells, a vital component of the innate immune system, are being explored for their therapeutic potential in OSCC due to their inherent ability to target and eliminate cancer cells without prior sensitization. This review uniquely focuses on the evolving role of NK cells specifically in OSCC, incorporating recent advancements in CAR-NK cell engineering and personalized therapy approaches that have not been comprehensively covered in previous reviews. The mechanisms through which NK cells exert cytotoxic effects on tumor cells include direct killing through the engagement of natural cytotoxic receptors and antibody-dependent cellular cytotoxicity (ADCC), making them promising agents in cancer immunotherapy. Additionally, the article explores recent advancements in engineering NK cells to enhance their antitumor activity, such as the modification with chimeric antigen receptors (CARs) to target specific tumor antigens. Clinical implications of NK cell-based therapies, including the challenges of integrating these treatments with existing protocols and the potential for personalized therapy, are examined. The review highlights the promise of NK cell therapies in improving outcomes for OSCC patients and outlines future directions for research in this dynamic field of oncological immunotherapy.

## Introduction

OSCC represents a common and formidable cancer in the head and neck area, marked by malignant growths arising from the squamous epithelium of the oral cavity (1–3). Occupying the sixteenth position worldwide in incidence and mortality rates, OSCC presents substantial public health challenges across various demographics (4). The oral cavity comprises multiple potential locales for these carcinomas’ emergence, encompassing the jaw’s mucosa, anterior tongue, posterior molars, mouth’s floor, hard palate, and the inner surfaces of the lips (3, 5).

Research indicates that over 90% of oral cancers manifest as squamous cell carcinomas, which underscores the predominant cellular origin of these tumors (2, 6). The genesis of OSCC involves multiple factors, with principal risk elements being the consumption of tobacco products, both smoked and smokeless, and the presence of high-risk strains of human papillomavirus (HPV) (7, 8). Notably, tobacco usage is strongly correlated with the development of OSCC, exhibiting a dose-response relationship where increased tobacco exposure elevates the risk of this malignancy (9). Furthermore, the prevalence of OSCC is notably affected by age, with individuals over the age of 40 facing a heightened risk, thereby underlining age as a significant demographic risk factor (10, 11). The global incidence of OSCC varies, with higher rates observed in areas where tobacco usage is widespread and in regions where socio-economic conditions hinder timely diagnosis and treatment (12). Despite advancements in diagnostic and therapeutic techniques, the prognosis for OSCC remains relatively dismal, especially for cases identified in advanced stages (12). Prompt detection and an integrated approach to treatment, combining surgery, radiation therapy, and chemotherapy, are imperative for enhancing survival outcomes.

Traditional treatment modalities for oral squamous cell carcinoma carry inherent limitations (**Table 1**) (3). Surgical interventions can cause significant trauma, impacting both the functionality and aesthetic appearance of the oral and maxillofacial regions (13, 14). Moreover, the concealed nature of primary tumor development often results in the imprecise identification of positive margins during surgical resection, potentially leaving residual cancerous cells (13). Chemotherapy may lead to adverse effects such as hair loss, nausea, vomiting, and increased susceptibility to infections (3). Similarly, radiotherapy can inflict temporary or permanent damage to the healthy tissues surrounding the cancer cells, markedly diminishing the patient’s quality of life (15, 16). Additionally, about one-third of patients continue to face the risks of recurrence and resistance to radiation and chemotherapy following these conventional treatments.

**Table 1:** Comparison of traditional treatment of OSCC.

Treatment	Advantages	Disadvantages
**Surgery**	Often completely removes the tumor; Immediate control the progress of OSCC; Can prevent further spread of cancer	Risk of significant side effects including disfigurement and loss of function; Requires hospitalization and recovery time; Not suitable for all stages or locations of tumors
**Radiation Therapy**	Non-invasive Can be targeted to minimize damage to surrounding tissues Effective for local control	Can cause long-term damage to surrounding healthy tissues; Side effects include dry mouth, sore throat, and potential for secondary cancers; Requires multiple sessions
**Chemotherapy**	Can target cancer cells throughout the body Can be combined with other treatments to improve outcomes	Systemic side effects including nausea, fatigue, hair loss; May not be effective against all cancer cells; Risk of developing resistance to drugs

Recent advancements have focused on enhancing the five-year survival rate of OSCC, which has improved from 59% to 70% over the period from 1990 to 2011 (5, 17). Nevertheless, survival outcomes are still significantly influenced by factors such as the stage of the tumor at diagnosis, its anatomical location, and the presence of regional or distant metastases (18–20). The management of OSCC is further complicated by its high rate of recurrence and the potential for developing secondary primary tumors, necessitating a comprehensive, multidisciplinary approach and continuous monitoring (6, 15, 21). A profound understanding of OSCC’s biological behavior and the molecular and cellular mechanisms underlying its development is crucial for the creation of targeted therapies that enhance clinical results (22, 23).

The aim of this review is to comprehensively explore and highlight the therapeutic potential of NK cells in the treatment of OSCC. The review will focus on the unique abilities of NK cells to target and eliminate cancer cells, particularly in the context of OSCC, and will cover recent advancements in NK cell engineering, such as the development of Chimeric Antigen Receptor NK (CAR-NK) cells. Thus, we conducted a comprehensive search in PubMed using the keywords “natural killer cells and Oral Squamous Cell Carcinoma,” covering all relevant studies published until July 2024. This search aimed to gather the latest research findings and developments in the role of natural killer (NK) cells in the context of Oral Squamous Cell Carcinoma (OSCC). It will also examine the integration of NK cell-based therapies with other treatment modalities, the challenges posed by the tumor microenvironment, and the future directions for research to enhance the clinical outcomes of NK cell therapies in OSCC.

## The OSCC tumor cells interactive with NK cells

The crosstalk between OSCC tumor cells and NK cells is pivotal for patient outcomes and disease progression (24). Since immune cells form the cellular foundation of immunotherapy, a profound understanding of immune infiltration within TME is essential to unravel the underlying molecular mechanisms and develop novel immunotherapeutic strategies to enhance clinical outcomes (25). Shao P et al. identified NK cell-associated genes, including SSNA1, TRIR, PAXX, DPP7, WDR34, EZR, PHLDA1, and ELOVL1, by quantifying NK cells and exploring their single-cell expression patterns in the HNSCC microenvironment. Their findings indicated that patients with high EZR expression might have a poor prognosis and worse clinical features. John S et al. conducted an immunohistochemical study to evaluate the distribution of cytotoxic T lymphocytes and natural killer cells in OSCC and oral epithelial dysplasia (OED) (26). The study aimed to assess the expression of CD8 and CD57 immune cells in OSCC, OED, and normal oral mucosa. OED with moderate or severe dysplasia and OSCC samples had higher levels of infiltrating immune cells, including T cells, B cells, NK cells, and macrophages, compared to normal mucosa. The results indicated that CD8 and CD57 expression increased from normal mucosa to OED, with the highest expression in OSCC. CD8 and CD57 could serve as surrogate markers to assess the malignant potential of lesions and determine the prognosis of patients with oral cancer (26).

Zhu et al. delves into the effects of oral cancer cell-derived exosomes (OcEXs) on the activity of NK cells, particularly their influence on NK cell receptors’ expression and functionality. Exosomes were extracted from oral cancer cell lines WSU-HN4 and SCC-9 via ultrafiltration, and their protein contents were analyzed using mass spectrometry, which identified a high concentration of transforming growth factor (TGF)-β1 (27, 28). Initial interactions with OcEXs resulted in the upregulation of activating receptors (NKG2D and NKp30) and downregulation of the inhibitory receptor (NKG2A) in NK cells, suggesting an enhancement in NK cell cytotoxicity. However, this expression waned over seven days, hinting at a potential induction of NK cell dysfunction over time. The cytotoxic capabilities of NK cells against oral cancer cells initially increased but declined following prolonged exposure to OcEXs. These findings underscore that while oral cancer-derived exosomes can temporarily boost NK cell activity, extended exposure leads to diminished cytotoxicity and functional impairment of NK cells. This investigation provides critical insights into the intricate interactions between cancer-derived exosomes and NK cells, presenting promising directions for advancing immunotherapy approaches in oral cancer (27).

Similarly, another study examines the influence of OCEXs on NK cell functions via the IRF-3 signaling pathway (29). This study provides profound insights into how exosomes from oral cancer cells can augment the cytotoxic capabilities of NK cells, an essential component of immune surveillance against tumors. Upon internalization by NK cells, the OCEXs facilitated increased NK cell proliferation and enhanced the release of cytotoxic molecules such as perforin and granzyme M, indicating a stimulatory effect on NK cells. The study identified NAP1, a protein highly concentrated in OCEXs, as pivotal in activating the IRF-3 pathway in NK cells. This activation bolstered the expression of IFN genes and chemokines, thereby enhancing NK cell functions. This mechanism not only deepens our understanding of cellular interactions within the tumor microenvironment but also indicates potential therapeutic targets for boosting NK cell activity against oral cancer (10). Yu X et al. investigated NUP62CL as an immunological and prognostic biomarker for OSCC (30). Tumor tissue samples from 319 OSCC patients, along with their clinical information, were retrospectively collected. The study identified high NUP62CL expression in OSCC tissues, which was associated with larger tumor size, advanced clinical stage, and poor prognosis. Additionally, NUP62CL protein expression was positively correlated with the abundance of CD3^+^CD4^+^ T cells, CD3^+^CD8^+^ T cells, CD56^+^ NK cells, CD68^+^CD86^+^ macrophages, and CD68^+^CD163^+^ macrophages, as well as immune checkpoints, including PD-1, PD-L1, and CTLA-4 protein expression. NUP62CL could serve as an effective prognostic and immunological biomarker for OSCC patients (30).

## Immunotherapy for OSCC

The expanding comprehension of TME in the context of immunotherapy for OSCC lays a vital groundwork for refining treatment modalities (1, 22). Particularly, the strategic modulation of NK cells within the TME uncovers promising avenues to boost the efficacy of immunotherapeutic interventions (31–33). Building upon these insights, the scope of immunotherapy for OSCC is broadening to encompass not only strategies centered on NK cells but also a diverse array of other immunotherapeutic agents (34). These advances strive to exploit the intricate interactions within the TME to enhance the precision and effectiveness of targeting and eradicating cancer cells (35). This evolving landscape offers renewed optimism for the development of more potent and tailored treatment options for patients with OSCC (36–38).

In the field of immunotherapy for OSCC, the TME is crucial, particularly affecting the efficacy of therapies that utilize NK cells (32, 34). Composed of a diverse assembly of cells, extracellular matrix elements, and signaling molecules, the TME frequently manifests an immunosuppressive influence that can impair the functionality of NK cells (24, 39). Nonetheless, the inherent capacity of NK cells to identify and annihilate malignant cells without the need for prior sensitization positions them as potent agents in the fight against OSCC (40). Recent breakthroughs in comprehending the dynamics between NK cells and the TME have catalyzed the formulation of approaches to amplify NK cell activity (35, 40). These include obstructing inhibitory signals within the TME and engineering NK cells to bear chimeric antigen receptors (CARs) (22, 24, 41). Such enhancements are designed to augment the natural cytotoxic abilities of NK cells, thus bolstering their capacity to effectively target and eliminate tumor cells in the formidable milieu of OSCC.

The immune system plays a pivotal role in combating cancer, involving both innate and adaptive immune responses. B and T cells are integral to adaptive immunity, while macrophages, eosinophils, NK cells, and dendritic cells (DCs) constitute the components of innate immunity (42–44). Cancer cells manipulate their surface antigen expression and suppress immune factor secretion, thereby evading and inhibiting immune-mediated destruction, fostering tumor progression (45). Advances in the understanding of NK cells, coupled with developments in immunology and genetic engineering technologies, have positioned NK cells as primary agents in cancer therapy (41). NK cells are increasingly recognized for their unique immunological responses, becoming pivotal figures in tumor immunotherapy (24). The development of OSCC is intricately linked to the immune microenvironment, making immunotherapy an increasingly utilized approach in this context (46). To elucidate the significance and potential of NK cell immunotherapy in the treatment of OSCC can provide a reference for its clinical application (47, 48).

Caruntu A et al. studied the persistent changes in peripheral blood lymphocyte subsets in patients with OSCC (49). They assessed the proportions of CD3^+^ total T lymphocytes, CD3^+^CD4^+^ helper T lymphocytes, CD3^+^CD8^+^ suppressor/cytotoxic T lymphocytes, CD3^-^CD19^+^ total B lymphocytes, and CD3^-^CD16^+^CD56^+^ NK cells in the peripheral blood of OSCC patients. The data, collected both pre- and post-therapy, indicated that the level of total CD3^+^ T lymphocytes in OSCC patients remained similar to that of control subjects, highlighting the stability of this immune parameter. However, pre-therapeutic data revealed a lower proportion of CD4^+^T, a significantly higher level of cytotoxic/suppressive CD8^+^T, and a much lower CD4^+^/CD8 T lymphocyte ratio compared to controls. In contrast, circulating NK CD16^+^ cells were markedly higher pre-therapy compared to the control group. These findings provide new insights into the immune alterations in the peripheral blood of OSCC patients, contributing to the understanding of the complex interplay between immuno-inflammatory processes and carcinogenesis (32, 49). While, Santos EM et al. evaluated the defense mechanisms of CD8^+^ and NK cells in oral and oropharyngeal squamous cell carcinoma (OSCC and OPSCC) (50). Fifty-four cases of squamous cell carcinoma (42 OSCC and 12 OPSCC) were treated immunohistochemically with CD8 and CD57 monoclonal antibodies. The study examined the relationship of CD8+ and NK cells with tumor size, lymph node metastasis (LNM), clinical staging (CS), overall survival (OS), and disease-free survival (DFS). Results showed that CD8 expression was higher in T1 and T2 tumors compared to T3 and T4 tumors, and in tumors without LNM and with CS II or III. However, there was no association between the biomarkers and OS or DFS. These findings suggest that the differential infiltration of CD8+ cells in OSCC and OPSCC may reflect a distinct tumor microenvironment with a favorable local cytotoxic immune response against neoplastic cells (50–52).

## NK cell activity is dependent on the balance between activating and inhibitory receptors on their surface

NK cells, a fundamental subset of innate lymphoid cells, originate in the bone marrow and reach maturity in secondary lymphoid tissues like the spleen, tonsils, and lymph nodes (53, 54). Distinguished from T cells by their lack of CD3 expression, NK cells are characterized by the presence of CD56 and CD16, which serve as definitive markers of their lineage (55). As pivotal agents in the immune system’s frontline defense, NK cells activate without prior sensitization, playing an essential role in protecting the body against pathogens and malignancies, notably against virally infected cells and tumors.

**Figure 1:**
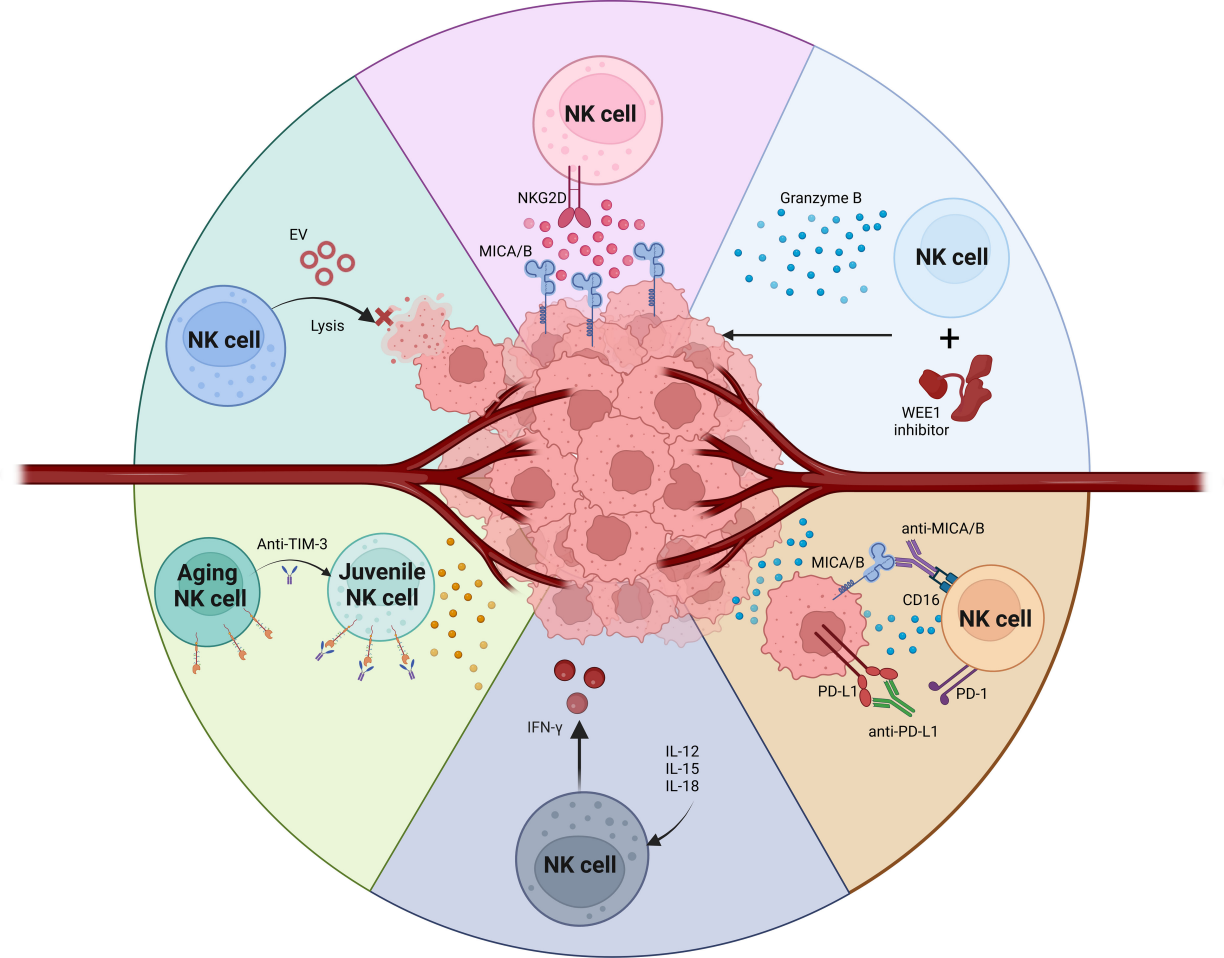
NK cells based therapy for OSCC tumor.

The primary activating receptors on NK cells include CD16, NKG2D, DNAM-1, and the natural cytotoxicity receptors (NCRs) (56, 57). These are counterbalanced by inhibitory receptors such as CD94, NKG2A, and a variety of killer immunoglobulin-like receptors (KIRs) (58). Additionally, NK cell functionality is modulated by checkpoint inhibitors like PD1, TIGIT, LAG3, and TIM3, which can dampen NK cell activity (41, 59, 60). These checkpoint inhibitors represent both a challenge and a critical target for therapeutic interventions. The efficacy of NK cells hinges on the intricate dynamics between these inhibitory and activating signals (61, 62). Harnessing these interactions is crucial for the advancement of NK cell-based immunotherapeutic strategies, particularly in the context of OSCC (46, 63). Enhancing NK cell activity in OSCC could significantly improve cancer management and patient outcomes, leveraging the potent capabilities of these immune cells to combat malignancy effectively (64) (**Figure 1**).

## The tumoricidal mechanism of NK cells against OSCC tumor cells

NK cells utilize a diverse array of mechanisms to eradicate tumor cells, playing a crucial role in the immune system’s defense against malignancies such as OSCC (65). One fundamental strategy is the “Missing-self” recognition, where NK cells target and destroy tumor cells that lack MHC-I molecules (66). This is accomplished through the release of cytotoxic molecules like granzymes and perforin (67). Upon forming an immune synapse with a target cell, NK cells orchestrate the reorganization of the actin cytoskeleton, facilitating the expulsion of perforin and granzymes (68). Perforin creates pores in the target cell membrane, enabling granzymes to penetrate and initiate apoptosis by cleaving cellular substrates (69). Additionally, NK cells can induce cell death via surface molecules such as Fas ligand (FasL) and TNF-related apoptosis-inducing ligand (TRAIL), which bind to their respective receptors on tumor cells, fostering apoptosis (55, 70). This not only aids in eliminating cancer cells but also promotes an inflammatory response that can attract further immune cells to the tumor site (71). Moreover, NK cells can trigger pyroptosis, a highly inflammatory form of cell death, through the activation of caspase 3 and gasdermin E, thus enhancing the immune clearance of tumor cells.

Another critical cytotoxic mechanism employed by NK cells is ADCC (72, 73). This process is facilitated by the high-affinity Fc receptor CD16 on NK cells, which interacts with antibodies bound to tumor cell antigens. The engagement of CD16 triggers the release of cytotoxic granules and pro-inflammatory cytokines, directly leading to the destruction of the target cells. ADCC is particularly significant in the context of cancer therapy, where monoclonal antibodies are designed to target specific tumor antigens (74). For instance, drugs like trastuzumab and rituximab target cancer cells for destruction via ADCC, with NK cells playing an essential role. Enhancing ADCC, whether by increasing CD16 expression or by improving NK cell affinity for antibodies, presents a promising avenue in cancer immunotherapy (75). By optimizing the natural capabilities of NK cells through ADCC, novel therapeutic strategies can be developed to improve the precision and effectiveness of tumor cell elimination, potentially enhancing outcomes in cancer treatment.

## NK cells related immunotherapy against oral squamous cell carcinoma

The research conducted by Gupta et al. explores the role of NK cells in monitoring and controlling OSCC, with a specific focus on the prognostic significance of histomorphological features (64). This study highlights the correlation between the presence of CD57 immunopositive NK cells and various markers of OSCC progression, including tumor budding, the size of tumor cell nests, and the lymphocytic response from the host. It demonstrates that increased NK cell activity is associated with favorable prognostic characteristics in OSCC, suggesting that the profiling of NK cells could inform therapeutic approaches and potentially act as indicators of OSCC progression. The findings advocate for the potential benefits of modulating NK cell activity to boost anti-tumor immune responses in OSCC, reinforcing the therapeutic promise of targeting these immune cells to enhance cancer treatment outcomes.

In oral cancer, circulating tumor cells (CTCs) deploy mechanisms that enable them to escape destruction by NK cells (76). A critical strategy involves the upregulation of the protein N-cadherin in self-seeded CTCs, which enhances their ability to evade immune detection. This upregulation induces functional exhaustion in NK cells through interactions with the KLRG1 receptor on NK cells. Self-seeded tumor cells, a subset of CTCs capable of returning to and proliferating within the primary tumors, exhibit elevated levels of N-cadherin. The soluble N-cadherin released from these cells engages the KLRG1 receptor on NK cells, leading to a state of exhaustion marked by diminished cytotoxicity and cytokine production. This interaction between N-cadherin and KLRG1 impairs NK cell functions, facilitating the circulation and seeding of new tumors by the tumor cells. Overexpression of N-cadherin in tumor cells not only increases their evasion from NK cell-mediated destruction but also enhances their seeding efficiency and potential for metastasis. Given these dynamics, targeting the N-cadherin/KLRG1 interaction emerges as a promising strategy to augment NK cell activity against oral cancer CTCs. By blocking N-cadherin or disrupting its interaction with KLRG1, it may be possible to restore NK cell functionality, potentially curtailing tumor metastasis and recurrence. This approach underscores the importance of understanding and manipulating key molecular interactions in the immune evasion strategies of tumor cells to develop more effective cancer therapies.

Researchers have discovered that these dysplastic cells frequently demonstrate aberrant activation of the Wnt/β-catenin pathway, primarily due to the overexpression of Wnt ligands that are dependent on Porcupine (PORCN) (77).The inhibition of PORCN, a key enzyme in the secretion of Wnt ligands, plays a critical role in the treatment strategy for OSCC, which often originates from potentially malignant lesions such as oral dysplasia. By pharmacologically targeting PORCN, the secretion of Wnt ligands is effectively inhibited, thereby reducing the activity of the Wnt/β-catenin pathway. The findings from this research suggest that targeting PORCN not only disrupts this critical signaling pathway but also significantly reduces the progression from oral dysplasia to OSCC. This points to a promising therapeutic strategy that aims to prevent the development of OSCC by intervening early in the cellular alterations within the oral cavity.

Despite advancements with immune checkpoint inhibitors (ICI) across various cancers, there is a pressing need for new strategies to broaden treatment efficacy, particularly for patients who do not develop effective antitumor T-cell responses. Radiation and pharmacological treatments can significantly alter the tumor immune microenvironment, prompting investigations into synergistic immunotherapeutic approaches. Patin EC et al. explored the enhancement of antitumor responses through the combined application of ataxia telangiectasia and Rad3-related kinase inhibition (ATRi) and radiotherapy (RT) (78). Utilizing the HPV-negative murine oral squamous cell carcinoma model, MOC2, we assessed the nature of the antitumor response post-ATRi/RT treatment through RNA sequencing and detailed flow cytometry analyses. The potential benefits of immunotherapies, particularly those targeting the T cell immunoreceptor with Ig and ITIM domains (TIGIT) and Programmed cell death protein 1 (PD-1) following ATRi/RT, were evaluated in the MOC2 model and corroborated in another model, SCC7. The results highlight that ATRi amplifies the inflammation induced by radiotherapy within the tumor microenvironment, with NK cells playing a pivotal role in enhancing treatment outcomes. It was demonstrated that the antitumor efficacy of NK cells could be significantly increased with ICI targeting TIGIT and PD-1. Analyses of clinical samples from patients receiving ATRi (ceralasertib) validate the translational potential of these preclinical findings (79). This study uncovers a previously unrecognized role of NK cells in the antitumor immune response to radiotherapy, which can be further augmented by leveraging small-molecule DNA damage-response inhibitors alongside immune checkpoint blockade, offering a novel avenue to enhance cancer therapy efficacy.

### CAR-NK cells for OSCC

Chimeric Antigen Receptor (CAR) NK cells refer to NK cells that have been genetically modified to express chimeric antigen receptors (CARs) on their surfaces (80, 81). CARs are synthetic proteins that consist of an extracellular antigen-binding domain, usually derived from a single-chain variable fragment (scFv) of an antibody, linked to an intracellular signaling domain of a T-cell receptor complex protein, such as CD3ζ. By introducing CARs into NK cells, researchers can redirect the specificity and activity of these cells to target specific antigens on OSCC tumor cells or other diseased cells. This approach has the potential to enhance the therapeutic efficacy of NK cells by making them more selective and potent against cancer or other diseases (81, 82). CAR-NK cells are currently being investigated as a potential treatment for various types of cancer and other diseases. They offer the advantage of being able to recognize and kill tumor cells directly, without the need for prior activation or antigen presentation by other immune cells (83). However, further research is needed to optimize the design and function of CAR-NK cells and to understand their safety and efficacy in clinical trials.

Jacobs MT et al. explored how memory-like differentiation, tumor-targeting monoclonal antibodies (mAbs), and chimeric antigen receptors (CARs) can enhance natural killer (NK) cell responses to head and neck squamous cell carcinoma (HNSCC) (84). To address this, the study hypothesized that memory-like (ML) NK cell differentiation, tumor targeting with cetuximab, and engineering with an anti-EphA2 CAR could improve NK cell responses against HNSCC. In this study, ML NK and conventional (cNK) cells from healthy donors were used. Cytokine production IFNγ, TNF, degranulate, and kill HNSCC cell lines and primary HNSCC cells were compated, both alone and in combination with cetuximab, *in vitro* and *in vivo* using xenograft models. Additionally, they engineered ML and cNK cells to express anti-EphA2 CAR-CD8A-41BB-CD3z and assessed their functional responses against HNSCC cell lines and primary tumor cells. Human ML NK cells exhibited enhanced production of IFNγ and TNF, as well as improved short- and long-term killing of HNSCC cell lines and primary targets compared to cNK cells. These responses were further enhanced by cetuximab. ML NK cells expressing anti-EphA2 CAR showed increased IFNγ production and cytotoxicity against EphA2+ cell lines and primary HNSCC targets compared to controls. These preclinical findings indicate that ML differentiation alone or combined with cetuximab-directed targeting or EphA2 CAR engineering can be effective against HNSCC. The results provide a strong rationale for investigating these combination approaches in early-phase clinical trials for patients with HNSCC (84, 85).

CAR-NK cells offer significant advantages in the treatment of OSCC, capitalizing on their derivation from a variety of sources such as peripheral blood, umbilical cord blood, and induced pluripotent stem cells (44, 53). This versatility and the potential for mass production address some of the challenges faced with patient-derived therapies. Notably, CAR-NK cells exhibit a safer risk profile, largely free from the severe toxicities often associated with CAR-T cell therapies, such as cytokine release syndrome (CRS) and immune effector cell-associated neurotoxicity syndrome (ICANS) (86). Additionally, they do not provoke graft-versus-host disease (GVHD), rendering them suitable for allogeneic use in clinical applications.

The capability of CAR-NK cells to effectively target and eliminate tumor cells, while navigating through and often overcoming the inhibitory mechanisms of the tumor microenvironment, positions them as a promising avenue for advancing treatment strategies in OSCC (41, 87, 88). Their lower incidence of severe toxicities and the absence of GVHD further underscore their potential as a transformative approach in oncology, offering a potent, scalable, and safer alternative to traditional CAR-T cell therapies (**Table 2**).

**Table 2:** Comparison of CAR-NK cells and CAR-T cells against OSCC.

Feature	CAR-NK Cells	CAR-T Cells	References
Source	Derived from peripheral blood, umbilical cord blood, Stem cell differentiation or cell lines	Derived from patient’s own T cells (autologous) or donor T cells (allogeneic)	(53, 89, 90)
Activation and Expansion	Can be activated and expanded ex vivo with cytokines (e.g., IL-2, IL-15)	Activated and expanded ex vivo using anti-CD3/CD28 beads and cytokines (e.g., IL-2)	(89, 91, 92)
Killing Mechanism	Direct cytotoxic effects through release of perforin and granzyme, and antibody-dependent cellular cytotoxicity (ADCC)	Direct cytotoxic effects through release of perforin and granzyme, and induction of apoptosis in target cells	(56, 89)
Advantages	Lower risk of cytokine release syndrome (CRS) and graft-versus-host disease (GVHD); Can target a broad range of tumors; Multiple NK cells sources.	Highly specific to target antigens; Proven efficacy in hematologic malignancies; Long-lasting persistence and memory formation.	(55, 89, 93, 94)
Disadvantages	Shorter lifespan and persistence *in vivo*; Potential for limited efficacy in solid tumors due to tumor microenvironment	Higher risk of CRS and GVHD; Complex and costly manufacturing process Potential for severe toxicities	(81, 89, 95, 96)
Current Research Status	Still in experimental stages, with ongoing research to optimize and validate their use in clinical settings.	Several FDA-approved therapies for hematologic cancers, with ongoing trials and research for efficacy in solid tumors, including OSCC	(83, 89, 97)

Utilizing CAR natural killer (CAR-NK) cells derived from induced pluripotent stem cells (iPSCs) and directed against MUC1, a protein commonly overexpressed in OTSCC (98). The efficacy of iPSC-derived MUC1-targeted CAR-NK cells was rigorously tested both *in vitro* and *in vivo*. MUC1 expression in OTSCC tissues and cell lines was verified through immunohistochemical and immunofluorescence analyses. Subsequent assessments demonstrated that these engineered NK cells could effectively target and annihilate MUC1-expressing cancer cells, exhibiting significantly enhanced cytotoxicity compared to iPSC-derived NK cells without the CAR modification. In a BNDG mouse xenograft model, the MUC1-targeted CAR-NK cells markedly curtailed tumor growth without causing substantial weight loss or hematological toxicity, indicating a favorable safety profile. The study suggests that MUC1-targeted CAR-NK cell therapy holds considerable promise as a new treatment modality for OTSCC, potentially offering higher efficacy and reduced toxicity compared to existing options. These findings advocate for the advancement to clinical trials to more comprehensively evaluate the effectiveness and safety of this innovative therapy in human subjects. This research not only underscores the potential of CAR-NK cell technology in treating solid tumors but also represents a significant stride forward in developing more effective therapies for patients with ad.

### Potential immuno-targets for NK cells related immunotherapy over OSCC

In the evolving landscape of immunotherapy for OSCC, identifying potential immune targets for NK cells presents a promising avenue for enhancing treatment efficacy (7). Among the key targets are the tumor associated antigens, like EGFR, which has been broadly used in clinical (99). Additional, stress-induced ligands MICA and MICB, which are recognized by the activating receptor NKG2D on NK cells. Overexpression of these ligands on OSCC cells can markedly enhance NK cell-mediated cytotoxicity. Furthermore, the blockade or modulation of inhibitory receptors such as PD1, TIGIT, TIM3, KIRs (Killer-cell Immunoglobulin-like Receptors) on NK cells, which interact with MHC class I molecules on tumor cells (100, 101). These molecules can restain NK cells via delivery immunosuppressive signals (53, 91). Exploring these targets within the tumor microenvironment of OSCC can lead to the development of targeted therapies that activate or enhance the innate cytotoxic responses of NK cells, offering a robust strategy to combat this challenging malignancy.

Utilizing a cytobrush providing a less traumatic alternative to traditional biopsies and can be executed without the need for specialized medical facilities. The samples are then analyzed using an advanced ELISA method, noted for its high sensitivity and specificity, facilitating the detection of specific biomarkers critical for early diagnosis of OSCC (102). The study targeted six biomarkers, including well-established ones like EGFR, p53, and Ki67 used in clinical diagnostics, alongside newer markers such as PD-L1, HLA-E, and B7-H6, which are pertinent to the tumor microenvironment and mechanisms of immune evasion. Implementing this novel diagnostic approach could become a pivotal tool for screening and early diagnosis, potentially decreasing the morbidity and mortality associated with OSCC. Its non-invasive nature, coupled with rapid processing times, positions it as an excellent option for routine monitoring and early intervention. This innovation marks a significant stride towards transforming current practices in the detection and management of oral cancer, making early diagnostics more accessible and effective.

The plasma levels of CASC15 are elevated in patients with stage I and II OSCC compared to those with oral ulcers and healthy controls, with no significant differences observed between the latter two groups (103). This upregulation of CASC15 effectively distinguishes OSCC patients from those with oral ulcers and healthy individuals. Further investigation revealed an inverse correlation between CASC15 and another LncRNA, MEG3, within OSCC tissues. Specifically, overexpression of CASC15 in OSCC cells led to a suppression of MEG3 expression, while overexpression of MEG3 did not affect CASC15 levels. The study suggests that CASC15 promotes the proliferation of OSCC cells by negatively regulating MEG3. These insights underscore the importance of CASC15 and MEG3 in the pathology of OSCC and highlight their potential as targets for therapeutic intervention (103).

#### Epidermal growth factor receptor

The study investigates the impact of cold atmospheric pressure plasma (CAP)-induced radicals on the epidermal growth factor receptor (EGFR), which is notably overexpressed in OSCC, aiming to understand the mechanism behind the selective cytotoxicity observed (104, 105). CAP treatment generates highly reactive radicals within both the plasma plume and the cell culture media. This results in a distinct selective killing effect on OSCC cells compared to normal human gingival fibroblasts. The selective cytotoxicity is specifically observed in OSCC cells that overexpress EGFR, where degradation and dysfunction of EGFR occur (105). This effect is absent in normal cells, highlighting the targeted action of CAP. Furthermore, the introduction of a nitric oxide scavenger prior to CAP treatment in the cell culture effectively mitigates the degradation and dysfunction of EGFR, as well as the associated cytotoxicity in OSCC cells (106). This evidence suggests that CAP could serve as a promising cancer treatment strategy by specifically inducing dysfunction in EGFR through nitric oxide radicals in OSCC cells that overexpress this receptor. This targeted approach offers potential for developing treatments that spare normal cells while effectively combating cancer cells, thereby improving therapeutic outcomes in oral squamous cell carcinoma (105).

The therapeutic efficacy of cetuximab in treating OSCC is significantly attributed to its ability to activate NK cells, thereby inducing ADCC and promoting cytokine secretion (107). Specifically, cetuximab-activated NK cells enhance dendritic cell (DC) maturation through the secretion of interferon-gamma (IFN-γ) (108). This process increases the cross-presentation of tumor antigens to CD8+ T cells, leading to the expansion of EGFR-specific T cells and bolstering the immune response against tumor cells (99). Moreover, this interaction between NK cells and DCs is characterized by bidirectional crosstalk, wherein increased DC expression further stimulates NK cell activation. This synergistic relationship enhances the overall immune response within the tumor microenvironment (109, 110). Research is ongoing to explore combinations of cetuximab with various drugs, such as IL-12, lenalidomide, monalizumab (an anti-NKG2A antibody), and the CD137/4-1BB agonist urea, to further augment the efficacy of cetuximab by boosting NK cell activation and the ADCC effect (108, 111, 112). These combinations aim to optimize cetuximab’s therapeutic potential in OSCC. Additionally, cetuximab treatment has been linked to increased expression of CTLA-4, TIM-3, and TGF-β on intratumoral regulatory T cells (Tregs) (113, 114). These changes may also play a role in NK-mediated DC maturation, contributing to a more robust immunological attack on tumor cells. This multifaceted impact underscores the complex interplay between various components of the immune system in cetuximab’s mechanism of action against OSCC, highlighting potential targets for enhancing treatment efficacy (115).

#### Boosting NKG2D activation signal for NK cells

HNSCC tumors tend to shed NKG2D ligands, leading to suppression of NK cells. Counteracting the effects of NKG2D ligand shedding to enhance NK cell activity includes apheresis of peripheral blood ligands (116), and antibody-mediated inhibition of MIC cleavage (117, 118). These studies highlight the significant role of the natural killer group 2D (NKG2D) receptors on NK cells and certain T cell subsets in the immunosurveillance of head and neck squamous cell carcinoma (HNSCC). These receptors are targeted by cancer evasion strategies, notably through the shedding of NKG2D ligands (NKG2DLs). Analysis of plasma and tumor samples from 44 HNSCC patients revealed that high levels of NKG2DLs in the plasma correlate with NK cell inhibition and disease progression. This finding was further substantiated by observations that NK cells are unable to infiltrate HNSCC tumors with high NKG2DL levels, suggesting a novel NKG2DL-dependent mechanism for tumor immune escape (119, 120). Moreover, the study also explores the potential of monitoring plasma NKG2DL levels for diagnostic and prognostic purposes, identifying patients who might benefit from therapies aimed at restoring NKG2D-dependent tumor immunosurveillance (121). Furthermore, experimental interventions in the study demonstrated that removing shed NKG2DLs (sNKG2DLs) from the plasma could restore NK cell function *in vitro* and enhance patient outcomes post-surgery. A proof-of-concept study involving adsorption apheresis to remove sNKG2DLs from plasma in rhesus monkeys was successful, suggesting this method could be a promising preconditioning strategy to boost the effectiveness of autologous and adoptive cellular cancer immunotherapies.

In another study, the role of CHMP2A in regulating tumor resistance to NK cell-mediated cytotoxicity was explored using a sophisticated “two cell type” whole-genome CRISPR-Cas9 screening system focused on human glioblastoma stem cells (GSC) and OSCC. The research identified CHMP2A as a pivotal regulator of GSC resistance to NK cell attacks, and these findings were further validated in a OSCC model (122). The investigation revealed that CHMP2A deletion in tumor cells activates the NF-κB pathway, which in turn enhances the secretion of chemokines. This increased chemokine production significantly boosts NK cell migration towards the tumor cells, thereby enhancing the immune response against the tumor. In the OSCC context, specifically within the CAL27 tumor model, it was demonstrated that CHMP2A mediates tumor resistance through a different mechanism-by the secretion of extracellular vesicles (EVs). These vesicles carry MICA/B and TRAIL, ligands known to induce apoptosis in NK cells, thus effectively reducing NK cell viability and inhibiting their anti-tumor functions. To substantiate these *in vitro* results, the study also included *in vivo* experiments where CHMP2A was deleted in CAL27 OSCC cells. This modification led to significantly increased NK cell-mediated killing in a xenograft model using immunodeficient mice, confirming the crucial role of CHMP2A in modulating tumor sensitivity to NK cell cytotoxicity. These findings highlight a complex mechanism of tumor immune escape facilitated by CHMP2A through the secretion of EVs that impair NK cell function. This study not only sheds light on the intricacies of tumor-immune system interactions but also identifies CHMP2A as a promising target for enhancing the efficacy of NK cell-based immunotherapies (122).

Using an immunocompetent mouse model and the syngeneic 4MOSC head and neck squamous cell carcinoma model, CHMP2A was knocked out (KO) via CRISPR/Cas9 in 4MOSC1 cells. These modified cells were then transplanted into immunocompetent hosts. The CHMP2A KO in 4MOSC1 cells enhanced NK cell-mediated tumor cell killing *in vitro*. Following transplantation, CHMP2A^KO^ in 4MOSC1 cells improved both T cell and NK cell antitumor activity compared to wild type tumors. There was no difference in tumor development between WT and CHMP2A KO tumors in immunodeficient mice. Mechanistically, CHMP2A KO tumors in immunocompetent mice showed increased CD4^+^ and CD8^+^ T cells, NK cells, and fewer myeloid-derived suppressor cells (MDSCs) (123). Thus, inhibiting CHMP2A and related pathways, it may be possible to counteract tumor resistance mechanisms and improve the therapeutic outcomes for patients suffering from various forms of cancer, including glioblastoma and head and neck squamous cell carcinoma (124).

#### Immune checkpoint blockade therapy

Immune checkpoint blockade therapy is a form of cancer immunotherapy that enhances the immune system’s ability to fight cancer by inhibiting the checkpoints that regulate immune responses (125). These checkpoints are often exploited by cancer cells to avoid being attacked by the immune system. Common targets of checkpoint inhibitors include the PD-1/PD-L1 and CTLA-4 pathways, which are crucial in maintaining immune homeostasis and preventing autoimmunity (125). By blocking these pathways, checkpoint inhibitors unleash the potential of T cells or NK cells to effectively recognize and destroy cancer cells (126). This approach has revolutionized the treatment of various cancers, including melanoma, lung cancer, and renal cell carcinoma, offering significant improvements in patient outcomes (127, 128). Despite its success, the therapy can also lead to immune-related adverse effects due to increased immune activity, necessitating careful management and monitoring.

CD38, a member of the ribosyl cyclase family, is expressed on various hematological cells and is known to contribute to immunosuppression and tumor promotion (129). While targeting CD38 with antibodies has been approved for treating multiple myeloma, its role in solid tumors like OSCC has not been extensively studied (130, 131).Ding Z et al. investigated the multifunctionality of CD38 in OSCC, focusing on its prognostic implications, immune balance, and interaction with immune checkpoints (132). This retrospective study analyzed 92 OSCC samples using immunohistochemistry (IHC) to determine the spatial distribution of CD38 and assess its diagnostic and prognostic value. Additionally, preoperative peripheral blood samples from 53 OSCC patients were analyzed via flow cytometry. The study also utilized the Tumor Immune Estimation Resource (TIMER) and cBioPortal databases to examine CD38 levels in various tumors and their correlation with the tumor immune microenvironment in HNSCC. CD38 was found ubiquitously in tumor cells (TCs), fibroblast-like cells (FLCs), and tumor-infiltrating lymphocytes (TILs). Patients with high CD38 expression in TCs (CD38(TCs)) had higher TNM stages and an increased risk of lymph node metastasis. Elevated CD38 in FLCs (CD38(FLCs)) was significantly associated with poor WPOI. Increased CD38 in TILs (CD38(TILs)) correlated with higher Ki-67 levels in tumor cells. Moreover, patients with high CD38(TCs) were more susceptible to postoperative metastasis, and those with high CD38(TILs) independently predicted shorter overall and disease-free survival. Interestingly, patients with high CD38(TILs), but not CD38(TCs) or CD38(FLCs), had significantly lower levels of CD3^+^CD4^+^T cells and a higher ratio of CD3^-^CD16^+^CD56^+^NK cells. This immune imbalance was linked to dysregulated immune checkpoint molecules (VISTA, PD-1, LAG-3, CTLA-4, TIGIT, GITR) and specific immune cell subsets, which were positively correlated with CD38 expression in HNSCC. CD38 is a poor prognostic biomarker for OSCC patients and plays a crucial role in modulating the immune microenvironment and maintaining circulating lymphocyte homeostasis (132). The co-expression of CD38 and immune checkpoint molecules offers new insights into immune checkpoint therapy (131).

##### Anti-PD-(L)1

Although most previous research has not demonstrated programmed cell death protein 1 (PD-1) induction on human NK cells, emerging studies have reported PD-1 expression under specific clinical conditions, including OSCC (133). This suggests a nuanced role for PD-1 in the context of NK cell function within certain tumors (134, 135). The activation of NK cells has been shown to significantly bolster the anti-tumor efficacy of PD-1/PD-L1 blocking antibodies in animal models. This enhancement indicates that effective NK cell activity could be crucial for optimizing PD-1-based immunotherapy in OSCC (136, 137). Enhancing NK cell function might involve disrupting their immunosuppressive interactions within the tumor microenvironment (TME), particularly with PD-1-expressing myeloid-derived suppressor cells (MDSCs), although direct interactions between these cells have not yet been documented (138). Additionally, the tumor response to NK cell-produced interferon-gamma (IFN-γ) includes upregulated expression of PD-L1, which can contribute to an immunosuppressive environment. Therefore, understanding and manipulating the dynamics of NK cell interactions and PD-1/PD-L1 pathways in the TME could provide significant therapeutic advantages in treating OSCC. This approach highlights the potential of integrating NK cell modulation into existing and developing immunotherapeutic strategies, aiming to enhance overall treatment outcomes (139).

##### Anti-TIGIT

TIGIT (T cell immunoreceptor with Ig and ITIM domains) is an inhibitory receptor expressed on NK cells, T cells, and T regulatory (Treg) cell subsets (79, 140). The signaling through TIGIT inhibits NK cell-mediated cytotoxicity and is linked to decreased cytokine production and degranulation capacity. Targeting TIGIT through antibody blockade has shown promising results *in vitro* and in animal models (141, 142). Blocking TIGIT reduces NK cell exhaustion, inhibits tumor growth, and enhances the production of proinflammatory cytokines by NK cells, indicating its potential as a therapeutic target to boost the immune response against tumors (141, 143).

##### Anti-TIM3

TIM3 (T cell immunoglobulin mucin-3) checkpoint inhibition is being explored as a treatment for advanced solid malignancies, including OSCC (144, 145). Previous studies, particularly in melanoma, have demonstrated that inhibiting TIM3 can alleviate NK cell exhaustion, enhancing their cytotoxic function (146). Several early-stage clinical trials (e.g., NCT02608268, NCT03744468) are currently investigating the efficacy of TIM3 inhibition in the treatment of advanced solid tumors, including OSCC. While the clinical effectiveness of these inhibitors has yet to be fully established, emerging evidence suggests that TIM3 plays a significant role in the progression of OSCC and may represent a valuable target for enhancing antitumor immunity (7).

##### Anti-LAG3

Inhibition of LAG3 using monoclonal antibodies has shown promising results in preclinical models. For instance, in a mouse model of OSCC, blocking LAG3 was able to limit tumor growth, suggesting that LAG3 could be a viable target for immunotherapy (147, 148). However, the precise contribution of NK cells in this context remains somewhat ambiguous. While NK cells are affected by LAG3 inhibition, LAG3 is also expressed on adaptive tumor-infiltrating lymphocytes, such as T cells, which are known to significantly influence tumor dynamics (144). The dual expression of LAG3 on both innate and adaptive immune cells complicates the interpretation of how LAG3 blockade benefits are mediated. Thus, while LAG3 blockade holds potential as a therapeutic strategy in treating advanced solid tumors, further research is necessary to disentangle the effects attributable to NK cells from those due to other immune cell types. This distinction is crucial for optimizing the therapeutic strategies targeting LAG3 and enhancing the overall effectiveness of cancer immunotherapy (125).

##### Anti-NKG2A

NKG2A is a receptor that carries an immunoreceptor tyrosine-based inhibitory motif (ITIM) and pairs with CD94 (111, 149). When NKG2A binds to its ligand HLA-E, it recruits the tyrosine phosphatase SHP-1, which subsequently suppresses NK cell-mediated cytotoxicity, including ADCC (150). *In vitro* studies have demonstrated that monalizumab can enhance the effector functions of both NK cells and CD8^+^ T cells. Its effect is found to be synergistic when used in combination with imrvalumab and cetuximab, enhancing overall immune response against tumors (111, 151). However, monalizumab alone does not effectively promote ADCC, but its combination with cetuximab significantly amplifies ADCC, pointing towards a synergistic approach to boost anti-tumor activity (152).

##### Anti-KIR inhibitory receptors

Lirilumab is a monoclonal antibody targeting KIR2D (killer-cell immunoglobulin-like receptors 2D), which are inhibitory receptors found on NK cells (153–155). These receptors normally interact with HLA-C on target cells to inhibit NK cell activity, thus restraining their cytotoxic response. Lirilumab binds to and blocks the activity of the inhibitory receptors KIR2DL1, KIR2DL2, and KIR2DL3 on peripheral NK cells, thereby reducing their inhibitory effects and enhancing NK cell anti-tumor response (153). This blockade also includes some interaction with KIR2DS1 and KIR2DL2, reducing their off-target effects. In clinical settings, particularly in patients with certain hematological malignancies, allogeneic transfer of NK cells lacking these inhibitory KIRs, facilitated by lirilumab, has shown potential in preventing relapse by amplifying the NK cells’ tumor-fighting capabilities (155, 156). These approaches illustrate the strategic targeting of NK cell inhibitory pathways as a means to potentiate their natural cytotoxic capabilities against cancer cells, offering promising enhancements to existing cancer immunotherapies (154).

##### Adenosine 2B receptor

Wang B et al. investigated the impact of co-inhibiting the adenosine 2b receptor (A2BR) and PD-L1 on the recruitment and cytotoxicity of NK cells in OSCC (157). Adenosine is known to modulate anti-tumor immune responses by affecting T-cells and NK cells within the tumor microenvironment (158, 159). However, the role of adenosine receptors in OSCC progression and their influence on immune checkpoint therapy is not well understood. In this study, tumor tissues from 80 OSCC patients admitted to Shandong University Qilu Hospital between February 2014 and December 2016 were analyzed. The expression of A2BR and PD-L1 in different regions of the tumor tissues, such as the tumor nest, border, and paracancer stroma, was detected using immunohistochemical staining. Treatment with BAY60-6583 increased PD-L1 expression in CAL-27 cells, an effect partially inhibited by PDTC, indicating that A2BR induces PD-L1 expression via the NF-κB signaling pathway. Furthermore, high A2BR expression in OSCC was linked to lower NK cell infiltration. Treatment with MRS-1706 (an A2BR inverse agonist) and/or a PD-L1-neutralizing antibody (CD274) enhanced NK cell recruitment and cytotoxicity against OSCC cells. Overall, the findings highlight the synergistic effect of co-inhibiting A2BR and PD-L1 in treating OSCC by modulating NK cell recruitment and cytotoxicity (157, 158).

Unlike T cells, NK cells are not restricted by major histocompatibility complex (MHC) molecules. Their activation is controlled through a balance of surface activating and inhibitory receptors. By blocking these immune checkpoints, such as TIGIT and TIM3, it is possible to prevent the immune escape of tumors, thereby enabling NK cells to more effectively exert their antitumor effects. This strategy aims to bolster the immune system’s natural ability to fight cancer, enhancing the efficacy of cancer immunotherapy (125, 160).

#### NK cells kill the OSCC cancer stem cells

The induced chemotherapy resistance and differentiation in oral cancer stem cells are associated with increased expression of CD54, B7H1, and MHC class I molecules (161). This process is mediated by a combination of membrane-bound or secreted IFN-γ and TNF-α from the NK cells. Interestingly, blocking these cytokines with specific antibodies to both IFN-γ and TNF-α, and not to each one alone, was necessary to inhibit the differentiation or resistance to NK cells. Furthermore, the use of these antibodies was required to prevent NK-mediated inhibition of stem cell growth, restoring their numbers to levels observed when the stem cells were cultured without anergized NK cells. The study also highlights that the effect of blocking IFN-γ, in the absence of TNF-α blocking, was particularly influential in preventing the increase in surface receptor expression, as adding an anti-IFN-γ antibody alone significantly reduced the upregulation of CD54, B7H1, and MHC class I. While antibodies to CD54 or LFA-1 did not inhibit differentiation, antibodies targeting MHC class I, but not B7H1, enhanced the cytotoxicity of NK cells against well-differentiated oral squamous carcinoma cells and OSCC that had differentiated following treatment with IL-2 and anti-CD16 monoclonal antibodies. Conversely, this approach inhibited the cytotoxicity of NK cells against undifferentiated OSCC. These findings suggest that NK cells, through their ability to kill or induce differentiation, may play a crucial role in preventing the progression of cancer by targeting cancer stem cells (161). This action could significantly impede cancer growth, invasion, and metastasis, offering potential therapeutic avenues for targeting cancer stem cells to control disease progression.

#### NK cell combine with icon immunotherapy against OSCC

Icon immunotherapy—a novel dual-targeting agent that focuses on both neovascular and cancer cell targets—in treating OSCC. The study used the human tongue cancer line TCA8113, both *in vitro* and *in vivo* within severe combined immunodeficiency (SCID) mice models (162). Icon, a chimeric immunoconjugate combining factor VII and human IgG1 Fc, was investigated for its potential to induce murine natural killer (NK) cell activity and activate the complement system to eradicate cancer cells. The results underscored the pivotal role of NK cells in mediating the cytotoxic effects of Icon. Further *in vivo* studies reinforced these findings. When tested on human tongue tumor xenografts in CB-17 strain of SCID mice—which possess normally functioning NK cells—Icon successfully eradicated the established tumors. Conversely, in SCID/Beige mice, which lack functional NK cells, Icon’s effectiveness was markedly reduced. This contrast highlights the essential role of NK cells in the therapeutic efficacy of Icon immunotherapy. The study concludes that NK cells are indispensable for the success of Icon immunotherapy in cancer treatment. The results also suggest that insufficient NK cell levels or activity could be a contributing factor to resistance against therapeutic antibodies, a finding that has implications for ongoing preclinical and clinical research into antibody-based therapies. This insight into the mechanism of Icon underscores the importance of NK cells and suggests that enhancing NK cell function could improve the outcomes of immunotherapeutic strategies targeting cancers like OSCC.

Jung EK et al. investigated the efficacy of natural killer (NK) cell therapy combined with chemoradiotherapy (CRT) in murine models of head and neck squamous cell carcinoma (HNSCC) (163). CRT successfully recruited mouse NK cells to the tumor site. Additionally, expanded and activated human NK cells (eNKs) were recruited to the tumor site in response to CRT, with CRT enhancing the anti-tumor activity of eNKs in an NOD/SCID IL-2Rγnull mouse model. Various HNSCC cell lines displayed different NK cell ligand activation patterns in response to CRT, which correlated with NK cell-mediated cytotoxicity. Identifying these activation patterns during CRT may improve patient selection for adjuvant NK cell immunotherapy combined with CRT. This study is the first to explore the antitumor function and recruitment of NK cells with CRT in an HNSCC mouse model (163), which provide evidence of major anti-tumor capacity of NK cells in solid tumor.

#### Inhibition myeloid-derived suppressor cells to augment the anti-tumor effects of NK cells

A particular focus is placed on the role of myeloid-derived suppressor cells (MDSCs) in inhibiting NK cell function within the tumor microenvironment of OSCC. Greene S, et al. explored the potential of NK-cell-based immunotherapy in overcoming limitations faced by T-cell-based therapies in treating OSCC (164). The research involves both murine models and human clinical samples to assess the suppressive actions of MDSCs derived from peripheral blood and tumor sites. In murine models, the study demonstrated that neutrophilic-MDSCs (PMN-MDSCs) expressing CXCR2 are pathologically accumulated in peripheral areas and within tumors, where they inhibit NK cell function through mechanisms such as TGFβ secretion and hydrogen peroxide production. A small-molecule inhibitor of CXCR1 and CXCR2, SX-682, was found to significantly reduce the accumulation of these suppressive cells in tumors. This facilitated enhanced infiltration, activation, and therapeutic efficacy of adoptively transferred murine NK cells. In the clinical setting, significant levels of circulating and tumor-infiltrating CXCR1/2+ PMN-MDSC and monocytic-MDSC were observed in patients with OSCC. These tumor-associated MDSCs displayed stronger immunosuppressive effects than their circulating counterparts, mediated through multiple independent mechanisms including TGFβ and nitric oxide. The findings suggest a promising therapeutic approach combining CXCR1/2 inhibitors with adoptively transferred NK cells, highlighting the need for clinical trials to evaluate this strategy’s efficacy in enhancing NK cell-mediated immunotherapy in OSCC (165).

While immune checkpoint inhibitors have revolutionized cancer treatment, their clinical benefits have been limited to a subset of patients (160, 166). Therefore, developing more effective methods to target tumor cells expressing immune checkpoint molecules is crucial (125). Fabian KP et al. studied the antitumor effects of PD-L1 targeting high-affinity natural killer (t-haNK) cells, which also target suppressive MDSC cells (167). For the first time, this study reports a novel NK cell line, PD-L1 targeting high-affinity natural killer (t-haNK) cells, derived from NK-92 cells. These cells were engineered to express high-affinity CD16, endoplasmic reticulum-retained interleukin (IL)-2, and a PD-L1-specific CAR. PD-L1 t-haNK cells retained the expression of native NK receptors and contained high levels of granzyme and perforin granules. Their results showed that PD-L1 t-haNK cells expressed PD-L1-targeting CAR and CD16, retained native NK receptors, and carried high levels of granzyme and perforin granules. Irradiated PD-L1 t-haNK cells were able to lyse all 20 human cancer cell lines tested, including triple-negative breast cancer (TNBC) and lung, urogenital, and gastric cancer cells. The cytotoxicity of PD-L1 t-haNK cells correlated with the PD-L1 expression on tumor targets and could be enhanced by pretreating the targets with interferon (IFN)-γ. *In vivo*, irradiated PD-L1 t-haNK cells inhibited the growth of engrafted TNBC and lung and bladder tumors in NSG mice. The combination of PD-L1 t-haNK cells with N-803 and anti-PD-1 antibody showed superior tumor growth control in engrafted oral cavity squamous carcinoma tumors in C57BL/6 mice. Additionally, when cocultured with human PBMCs, PD-L1 t-haNK cells preferentially lysed the MDSC population without affecting other immune cell types (167).

#### Cytokine and chemokine signal pathway enhance NK cell based therapy against OSCC

The chemokine and cytokine signal have received significant attention due to their role in cancer development (168). Liu H et al. explored the effects of adenovirus-mediated overexpression of interleukin-21 (IL-21) on the development of OSCC *in vitro (* 169). In tumor cells, IL-21 enhances the immune response by increasing the cytotoxic activity of natural killer cells, B cells, and CD8^+^ T cells, leading to tumor cell apoptosis. The therapeutic effects of IL-21 have been studied in various diseases, with numerous clinical trials underway (168). This study was to determine the role of IL-21 in OSCC *in vitro*. IL-21 expression in OSCC tissues was detected using RT-qPCR, western blotting, and immunohistochemistry analyses, which revealed decreased IL-21 protein expression in OSCC tissues. IL-21 was overexpressed in CAL-27 cells using adenovirus. IL-21 overexpression inhibited OSCC cancer cell proliferation. Additionally, wound healing assays indicated that IL-21 overexpression suppressed cell migration, while TUNEL staining and flow cytometry analysis demonstrated that IL-21 overexpression promoted cell apoptosis via activation of the JNK signaling pathway. These findings suggest that IL-21 may serve as a potent antitumor agent in OSCC (169).

Upregulation of CCL2/CCR2 is linked to cancer progression, metastasis, and relapse (168, 170). By integrating scRNA-seq data with TCGA data, we discovered that the IL6/IL6R and CCL2/CCR2 signaling pathways have a more significant impact on immune evasion by NK cells in the HPV-negative HNSCC cohort compared to the HPV-positive cohort. In orthotopic mouse models, blocking IL6 with a neutralizing antibody suppressed HPV-negative tumors but not HPV-positive ones, and this suppression was accompanied by increased infiltration and proliferation of CD161^+^ NK cells. Notably, combining the CCR2 chemokine receptor antagonist RS504393 with IL6 blockade resulted in a more pronounced antitumor effect, characterized by more activated intratumoral NK cells in HPV-negative HNSCC compared to either agent alone. These findings demonstrate that dual blockade of the IL6 and CCR2 pathways effectively enhances NK cell-mediated antitumor activity in HPV-negative HNSCC, offering a novel strategy for treating this type of cancer (171).

Another study from Crist M et al. investigated how metformin enhances natural killer (NK) cell functions in HNSCC by inhibiting CXCL1 (172). This study included results from two phase I open-label trials involving HNSCC patients treated with metformin (NCT02325401, NCT02083692). Peripheral blood samples were collected from patients before and after metformin treatment or from newly diagnosed HNSCC patients. NK cells were treated with either a vehicle or metformin and then analyzed by RNA sequencing (RNA-seq). Significant pathways identified by RNA-seq were inhibited, and NK cells were further analyzed using NKCA, ELISA, and western blot analyses. The study found increased activated peripheral NK cell populations in patients treated with metformin and enhanced NK cell tumor infiltration in preoperatively treated HNSCC patients. Metformin increased the production of antitumorigenic cytokines ex vivo, particularly perforin. It also enhanced NK cell cytotoxicity against HNSCC cells, inhibited the CXCL1 pathway, and stimulated the STAT1 pathway. Exogenous CXCL1 was found to prevent metformin-enhanced NK cell-mediated cytotoxicity. Metformin-mediated NK cell cytotoxicity was independent of AMP-activated protein kinase but dependent on both the mechanistic target of rapamycin and pSTAT1. These findings reveal a new role for metformin in promoting immune antitumorigenic function through NK cell-mediated cytotoxicity and CXCL1 downregulation in HNSCC, informing future immunomodulating therapies in this context.

#### Reduce the epithelial-mesenchymal transition

Epithelial–mesenchymal transition (EMT) is a critical process in embryonic development, fibrosis, and cancer invasion (173). Despite recent advances in treatment, the 5-year overall survival rate of oral squamous cell carcinoma (OSCC) has not improved. EMT plays a significant role in the local recurrence and lymph node metastasis of oral cancer. Wang C et al. studied how heparanase (HPSE) promotes malignant characteristics in human oral squamous carcinoma cells by regulating EMT-related molecules and levels of infiltrating NK cells (174). Knocking down HPSE expression reduced the proliferation rate of SCC-25 cells, leading to a significant increase in the percentage of cells in the G0/G1 phase, and suppressed cell migration and invasion. E-cadherin mRNA and protein expression increased, while Snail and Vimentin expression decreased. RNA sequencing between the small interfering RNA and negative control groups identified 42 differentially expressed genes, including syndecan binding protein, RAB11A (a member of the RAS oncogene family), and DDB1 and CUL4-associated factor 15. These findings indicate that HPSE knockdown suppresses SCC-25 cell proliferation, invasion, migration, and EMT, potentially through syndecan binding protein and RAB11A. Additionally, HPSE may regulate the activation levels of infiltrating NK cells, possibly via DDB1 and CUL4-associated factor 15.

#### Modulate the RNA-network combine with NK cell therapy

The networks involving circle RNAs (circRNAs) and small RNAs can impact numerous molecular targets, driving specific cellular responses and determining cell fates. In cancer, ncRNAs have been identified as oncogenic drivers and tumor suppressors across all major cancer types (175).. Liu L et al. studied the role of SP2-induced circPUM1 in modulating chemoresistance and natural killer (NK) cell toxicity in OSCC (176). Previous research has established that NAP1L1 plays critical roles in various cancers and is involved in chemoresistance in hepatocellular carcinoma and glioma. The study identified NAP1L1 as a downstream target of miR-770-5p and crucial for circPUM1-mediated chemoresistance and NK cell toxicity in OSCC cells (175, 177). circPUM1 in OSCC cells and generated dysregulated circPUM1 cell models, demonstrating that circPUM1 promotes chemoresistance and NK cell toxicity. Furthermore, the transcription factor SP2 regulates circPUM1 expression in OSCC cells, with circPUM1 acting as a molecular sponge for miR-770-5p. NAP1L1, a downstream target of miR-770-5p, is essential for circPUM1-mediated cisplatin resistance and NK cell cytotoxicity in OSCC cells. The network comprising SP2, circPUM1, miR-770-5p, and NAP1L1 presents a promising avenue for developing novel diagnostic or therapeutic targets for OSCC. NAP1L1 overexpression promoted cell viability, which was reduced by downregulated circPUM1, and NAP1L1 downregulation alleviated the increased cell viability promoted by upregulated circPUM1 in cisplatin-treated OSCC cells. These findings suggest that NAP1L1 plays a crucial role in circPUM1-mediated chemoresistance and NK cell toxicity in OSCC cells (178).

#### Tertiary lymphoid structures

Tertiary lymphoid structures (TLS) are ectopic lymphoid structures in cancers typically associated with favorable prognosis, but their prognostic significance in OSCC is not well understood, and the relationship between TILs and TLSs in OSCC has been seldom explored (179, 180). Li Q et al. investigated the prognostic value of TLS andTILs in OSCC (181). In this study, markers associated with TLS, including peripheral node addressin (PNAd) in high endothelial venules, CD20 in B cells, and CD3 in T cells, were examined in 168 OSCC patients. Survival analysis was conducted to compare TLS-positive and TLS-negative cohorts. Additionally, TILs were identified by staining CD8^+^ cytotoxic T cells and CD57^+^ NK cells. TLSs were found to be highly organized structures in 45 (26.8%) cases. Patients with TLS-positive tumors had significantly better 5-year overall survival (OS) rates (88.9% vs. 56.1%, P < 0.001) and relapse-free survival (RFS) rates (88.9% vs. 63.4%, P = 0.002). The presence of TLS was identified as an independent prognostic factor for both 5-year OS (hazard ratio [HR] = 3.784; 95% confidence interval [CI], 1.498-9.562) and RFS (HR = 3.296; 95% CI, 1.279-8.490) in multivariate analysis. Additionally, a higher density of CD8+ T cells and CD57^+^ NK cells was observed in TLS-positive sections compared to TLS-negative ones (P < 0.001), and their combination provided higher predictive accuracy (AUC = 0.730; 95% CI, 0.654-0.805). In conclusion, the results suggest that TLS is an independent positive prognostic factor for OSCC patients, providing a theoretical basis for the future diagnostic and therapeutic value of TLS in OSCC treatment.

### Limitations of NK cells against OSCC

While NK cell-based immunotherapies offer promising potential in treating OSCC, several limitations and challenges must be addressed to improve their efficacy (86, 182).

#### Tumor microenvironment challenges

The TME in OSCC is rich in immunosuppressive factors such as TGF-β, IL-10, and myeloid-derived suppressor cells (MDSCs). These factors inhibit NK cell activation and function, reducing their ability to attack tumor cells effectively. Additionally, the dense extracellular matrix (ECM) and stromal components in the TME physically impede NK cell infiltration and migration to the tumor site, limiting their cytotoxic effects on cancer cells.

#### Intrinsic limitations of NK cells

Heterogeneity and Variability. NK cells are not a homogeneous population, and their activity can vary significantly between individuals. This variability affects the consistency and predictability of NK cell-based therapies.

#### Short lifespan and persistence

NK cells have a relatively short lifespan in the bloodstream, and maintaining their persistence and activity within the TME is challenging. This necessitates repeated infusions or genetic modifications to enhance their longevity and efficacy.

#### Therapeutic delivery challenges

Targeting and Specificity: Ensuring that engineered NK cells, such as CAR-NK cells, specifically target OSCC cells without affecting normal tissues remains a significant challenge. Off-target effects can lead to unintended damage to healthy cells and tissues.

#### Resistance mechanisms

Tumor cells can develop mechanisms to evade NK cell detection, such as downregulating stress ligands or upregulating inhibitory signals that prevent NK cell activation. This adaptive resistance can reduce the overall effectiveness of NK cell therapies.

#### Clinical and practical limitations

Cost and Complexity: The production and administration of NK cell-based therapies are complex and costly. This includes the isolation, expansion, and genetic modification of NK cells, which require specialized facilities and expertise.

#### Regulatory hurdles

NK cell therapies are relatively new, and regulatory approval processes can be lengthy and stringent. Ensuring safety, efficacy, and quality control for these therapies adds to the development timeline and cost.

Nevertheless, overcoming the limitations of NK cells against OSCC has become a significant focus in cancer research. Integrating NK cell-based therapies with other treatments, such as immune checkpoint inhibitors, can significantly enhance their efficacy. Modulating the tumor microenvironment to be more favorable to NK cell activity is another promising approach (183, 184). Furthermore, advancements in genetic engineering, such as the development of CAR-NK cells, can improve targeting and persistence. Additionally, modifying NK cells to express cytokines or receptors that boost their activity within the tumor microenvironment can address some inherent limitations. Ongoing research and innovative strategies are crucial for overcoming these barriers and optimizing NK cell therapies for superior clinical outcomes.

### Future outlook

The field of immunotherapy for OSCC has evolved dramatically, with Natural Killer (NK) cell-based therapies emerging as a promising frontier. NK cells, a critical component of the innate immune system, possess inherent cytotoxic abilities that can be harnessed and enhanced to target cancer cells. As research delves deeper into the unique properties of NK cells, their role in combating OSCC is being redefined, offering new therapeutic avenues and hope for improved patient outcomes. NK cells exhibit a natural ability to detect and kill cells undergoing stress, such as cancerous cells, without the need for prior sensitization. This capability makes them an attractive option for immunotherapy, particularly in OSCC, where the early detection and treatment of cancer cells are crucial for improving survival rates.

Furthermore, NK cells’ mechanisms of action do not rely on antigen presentation by Major Histocompatibility Complex (MHC) molecules, which are often downregulated in OSCC cells to evade immune detection. This allows NK cells to overcome one of the primary mechanisms of immune escape utilized by tumor cells. Recent advances in biotechnology have enabled the engineering of NK cells to enhance their anticancer activity. CAR can be expressed on NK cells, creating CAR-NK cells that combine the specificity of antibody-based recognition with the potent cytotoxic activity of NK cells. These engineered NK cells can be directed to target specific antigens expressed on the surface of OSCC cells, increasing their efficacy and specificity. Clinical trials involving CAR-NK cells have shown promising results, indicating significant potential for their use as a treatment modality for OSCC.

However, challenges remain in the clinical application of NK cell therapies. One of the main hurdles is the immunosuppressive tumor microenvironment (TME) of OSCC, which can inhibit NK cell function. Strategies to overcome this include the use of adjuvant therapies that modify the TME to be more conducive to NK cell activity. For instance, combining NK cell therapy with checkpoint inhibitors or modulators of the TME can enhance the effectiveness of NK cells. Looking forward, the integration of NK cell-based therapies into the standard care for OSCC appears promising. Ongoing research aims to optimize the delivery, specificity, and persistence of NK cells within the TME. Furthermore, as our understanding of the molecular and cellular interactions within the OSCC TME improves, so too will strategies for enhancing NK cell function.

The ultimate goal is to develop a tailored immunotherapy approach that can be integrated with existing surgical and chemotherapeutic treatments to provide a comprehensive treatment strategy for OSCC patients. In conclusion, NK cell-based immunotherapy holds a bright future in the management of OSCC. Continued research and clinical trials are essential to harness the full potential of NK cells, refine their application, and solidify their place in the oncological arsenal against oral cancer.
